# The Preparation, Characterization, and Pressure-Influenced Dihydrogen Interactions of Tetramethylphosphonium Borohydride

**DOI:** 10.3390/ma16155334

**Published:** 2023-07-29

**Authors:** Tomasz Jaroń

**Affiliations:** Faculty of Chemistry, University of Warsaw, Pasteura 1, 02-089 Warsaw, Poland; tjaron@uw.edu.pl

**Keywords:** borohydrides, tetramethylphosphonium, crystal structure, high pressure chemistry, dihydrogen interactions

## Abstract

Tetramethylphosphonium borohydride was synthesized via an ion metathesis reaction in a weakly-coordinating aprotic environment. [(CH_3_)_4_P]BH_4_, in contrast to related [(CH_3_)_4_N]^+^ compounds which tend to crystallize in a tetragonal system, adopts the distorted wurtzite structure (*P*6_3_mc), resembling some salts containing analogous ions of As and Sb. [(CH_3_)_4_P]BH_4_ decomposes thermally in several endo- and exothermic steps above ca. 240 °C. This renders it more stable than [(CH_3_)_4_N]BH_4_, with a lowered temperature of decomposition onset by ca. 20 °C and solely exothermic processes observed. Raman spectra measured at the 0–10 GPa range indicate that a polymorphic transition occurs within 0.53–1.86 GPa, which is further confirmed by the periodic DFT calculations. The latter suggests a phase transition around 0.8 GPa to a high-pressure phase of [(CH_3_)_4_N]BH_4_. The *P*6_3_mc phase seems to be destabilized under high pressure by relatively closer dihydrogen interactions, including the C–H…H–C contacts.

## 1. Introduction

In about the last two decades, borohydrides have become one of the most intensely investigated groups of materials [1,2]. This has been motivated mainly by a high gravimetric content of hydrogen in these compounds, rendering some of them prospective for onboard hydrogen storage for fuel cell vehicles. Obviously, high hydrogen content alone (>6.5 wt%) is not sufficient for a material to fulfill the economically-based requirements listed by the U.S. Department of Energy [3]. Besides their minimal capacity, the other parameters also remain crucial for potential applicability, especially the temperature of H_2_ evolution (preferably not very far from 100 °C) and the high purity of the released gas.

In order to meet the required parameters of hydrogen storage, various simple and complex borohydrides have been screened, stimulating the development of synthetic capabilities [4]. In this context, borohydrides based on organic cations have received significant interest as precursors for the synthesis of various borohydride complexes [5,6,7,8]. Such organic borohydrides, typically applied as reducing agents [9], are soluble in a wide variety of solvents, which facilitates their use in salt metathesis reactions.

Tetraorgano-phosphonium compounds tend to be more thermally stable in comparison to their ammonium counterparts [10], which may be useful for the stabilization of synthetic precursors [5] or in other potential applications. On the other hand, the hydrogen atoms closest to the phosphorous atom are significantly more acidic in tetraalkylphosphonium cations than those in their ammonium analogues [11]. It would be interesting to establish whether this feature could promote the hydrogen evolution from tetraalkylphosphonium borohydrides under elevated temperatures due to interaction of hydridic (B–H^δ−^) and weakly protic (P–C–H^δ+^) hydrogen atoms. Significantly elevated pressure, bringing the B–H…H–C distances expected for the solid-state structure of these materials closer together, could reveal a similar destabilizing effect. Such tetraalkylphosphonium borohydrides have not been reported before, contrary to tetraphenylphosphonium borohydride, which has already been utilized for the preparation of numerous borohydride complexes [5,6,8,12,13,14].

To elucidate their structural features and to check their stability under high temperatures or pressurization, we focused on the simplest compound, tetramethylphosphonium borohydride, [(CH_3_)_4_P]BH_4_. We prepared this material and characterized it thoroughly using several analytical techniques, allowing for confirmation of its identity and purity [15,16,17]. We discuss its crystal structure and thermal decomposition and compare the high-pressure behavior of this compound to its lighter analogue, [(CH_3_)_4_N]BH_4_. This is achieved by Raman spectroscopy measurements for the sample compressed in a diamond anvil cell to >10 GPa and theoretical calculations utilizing DFT formalism for periodic systems.

## 2. Materials and Methods

All of the manipulations were performed in an inert gas (Ar) atmosphere, either in Schlenk-type glassware or in a glovebox (<1 ppm O_2_, <1 ppm H_2_O); the latter was also used for storage of the reagents and products. The synthetic procedures are described below.

### 2.1. Mechanochemical Attempt at Synthesis

[(CH_3_)_4_P]Br, 3 mmol, and LiBH_4_, 3.06 mmol, 102% (both Sigma-Aldrich, St. Louis, MO, USA) were weighted, mixed, and placed in a stainless steel vessel sealed under Ar. A Testchem laboratory vibrational mill (LMW-S) was used for milling, with the nominal frequency of 1400 rpm. The mechanochemical reaction was carried out for 40 min in 5-min periods alternated with several minutes rest to avoid overheating and thermal decomposition of the products. The vessel was cooled with liquid nitrogen to maintain it at room temperature.

### 2.2. Synthesis of [(CH_3_)_4_P][Al(OC(CF_3_)_3_)_4_]

The mixture of water and acetone (ca. 2:1) was used as a solvent, similar to the ref. [18]. A solution of 1.72 mmol of (CH_3_)_4_PBr in 9 mL of this solvent was added to the solution of 1.72 mmol of Li[Al(OC(CF_3_)_3_)_4_] (synthesized according to ref. [19]) in ca. 27 mL of the solvent. The mixture was stirred in an open container for ca. 24 h at 40 °C to allow for slow evaporation of the acetone and crystallization of the product poorly soluble in water (isolated with ca. 80% yield).

### 2.3. Solvent-Mediated Synthesis of [(CH_3_)_4_P]BH_4_

The reaction was performed using an H-vessel equipped with a glass frit (no. 4) [20]. Approximately 0.84 mmol (105%) of [(*n*-C_4_H_9_)_4_N]BH_4_ dissolved in 5 mL dichloromethane (DCM) was added to 0.8 mmol of [(CH_3_)_4_P][Al(OC(CF_3_)_3_)_4_] suspended in DCM (12 mL). The mixture was stirred for 30 min while the low-density product gathered on the top of the solution. The by-product, [(*n*-C_4_H_9_)_4_N][Al(OC(CF_3_)_3_)_4_], was extracted using five cycles of filtration and distillation, followed by evaporation of the solvent.

### 2.4. Analytical Techniques and Data Processing

Single crystals of [(CH_3_)_4_P]BH_4_ were grown from anhydrous acetonitrile solution via slow evaporation of the solvent. The crystals were covered with Krytox 1531 oil and mounted on a goniometer using a nylon loop. The measurements of single-crystal diffraction were performed using an Agilent Supernova X-ray diffractometer equipped with a CuKα radiation micro-source. CrysAlisPro software (v. 38.43) [21] was used for data reduction, while SHELXT [22] and Olex2 [23] were utilized for the structure solution and refinement, respectively. The B–H and C–H were restrained at 1.190(10) Å and 1.083(10) Å, respectively, and all of the hydrogen atoms were refined with fixed displacement parameters, [U(H) = 1.5U_eq_(C or B)]. For the structure measured at 300 K and for [(CH_3_)_4_P]Cl as well, several DANG restraints involving hydrogen atoms were applied. An Agilent Supernova X-ray diffractometer (CuKα radiation micro-source) and Panalytical X’Pert Pro diffractometer (parallel beam; CoKα radiation) were used to measure the powder diffraction patterns (PXDs). Jana2020 [24] was utilized for Rietveld refinement of the sample of [(CH_3_)_4_P]BH_4_ powder. If not stated otherwise, all of the geometrical parameters mentioned in the text are related to the single-crystal structures measured at 100 K due to their best achievable quality.

CCDC entries 2271774–2271777 contain the supplementary crystallographic data for this paper. These data can be obtained free of charge from the Cambridge Crystallographic Data Centre.

Raman spectra were measured using a Horiba T64000 Raman spectrometer from Jobin Yvon equipped with a Si CCD detector. A laser of 532 nm wavelength was used, and the spectra were collected at room temperature under a microscope using a 10× objective. The samples were sealed in 1 mm Ar-filled quartz capillaries or placed in a diamond anvil cell (DAC). A Diacell^®^ One20DAC from Almax (Diksmuide, Belgium) equipped with type IIa Boehler-Almax diamond Raman-grade anvils with 500 μm culets was used. Stainless steel gaskets were used with a hole of ca. 250 μm drilled after indentation. Krytox 1531 oil was used as a pressure-transmitting medium, and the crystals of the samples were loaded together with the small scraps of ruby necessary for pressure calibration.

TGA/DSC measurements were performed using a Netzsch STA 409 PG instrument. The samples of ca. 10 mg were heated from 20 °C to 450 °C (10 °C min^−1^ heating rate) in Al_2_O_3_ crucibles under Ar flow (100 mL min^−1^). The results were processed using Netzsch Proteus^®^ software v. 4.8.4. The onset decomposition temperature was estimated as an intersection of the two trend lines approximating linear parts of the TGA curve before and during decomposition.

Density functional theory (DFT) calculations for the periodic systems were carried out using CASTEP v. 2017 (Biovia, San Diego, CA, USA) [25]. Several structural models of [(CH_3_)_4_P]BH_4_ were derived from the experimental data as described below. Generalized gradient approximation (GGA) was used with PBE functional and Tkatchenko–Scheffler dispersive correction [26]. The cut-off value of 600 eV was applied to achieve good energy convergence. The density of the k-point grid was set below 0.05 Å^−1^, and ultrasoft, generated on the fly pseudopotentials, were used to obtain more accurate lattice parameters. The unit cells were optimized under the given hydrostatic pressure using the BFGS algorithm. The Birch–Murnaghan equations of state (EoS) were fitted to the theoretical data using EoSFit software v. 7.60 [27,28].

## 3. Results and Discussion

### 3.1. Synthesis

Due to the low solubility of tetramethylphosphonium bromide in water and the decomposition of borohydrides occurring in the usual protic solvents, we attempted to carry out the mechanochemical synthesis of [(CH_3_)_4_P]BH_4_ according to Equation (1):

[(CH_3_)_4_P]Br + LiBH_4_ → [(CH_3_)_4_P]BH_4_ + LiBr (1)

**Figure 1:**
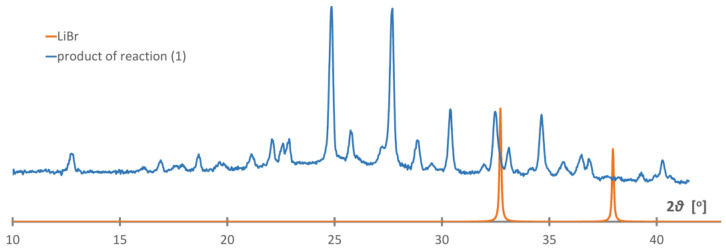
PXD pattern of the products of the reaction attempted according to Equation (1), blue curve, compared to the pattern generated for LiBr, orange curve (Co Kα).

While the mechanochemical approach is very convenient for the study of a broad range of chemical compounds [29], it appeared that the reaction (1) did not occur according to our expectations, leading to unknown products and lacking the LiBr by-product in the post-reaction mixture, Figure 1. Apparently, the difference in lattice energies between the reagents and products may not be sufficient to perform this salt metathesis reaction without a solvent. As the diffraction pattern does not contain any signals of the reagents, an addition reaction may have occurred with the formation of mixed-ion salt. Unfortunately, the indexation of the PXD pattern appeared unsuccessful; therefore, we skipped further identification of this product.

As the mechanochemical approach appeared not to be applicable in this case, we utilized a more universal path of synthesis, allowing for the preparation of a broad range of ionic compounds [5]. First, the [(CH_3_)_4_P][Al(OC(CF_3_)_3_)_4_] precursor was prepared via the modified procedure for the synthesis of its ammonium analogue:

[(CH_3_)_4_P]Br + Li[Al(OC(CF_3_)_3_)_4_] → [(CH_3_)_4_P][Al(OC(CF_3_)_3_)_4_]↓ + LiBr (2)

Subsequently, the precursor formed in reaction (2) was used for the salt metathesis reaction, leading to the desired product:

[(CH_3_)_4_P][Al(OC(CF_3_)_3_)_4_] + [(*n*-C_4_H_9_)_4_N]BH_4_ → [(CH_3_)_4_P]BH_4_↓ + [(*n*-C_4_H_9_)_4_N][Al(OC(CF_3_)_3_)_4_] (3)

**Figure 2:**
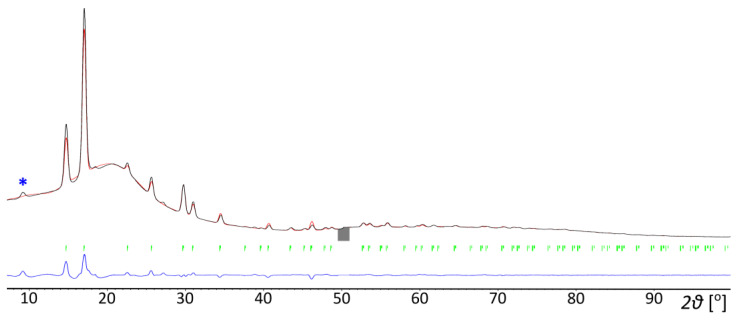
Rietveld refinement profile of [(CH_3_)_4_P]BH_4_ prepared in reaction (3). The strongest signal of the [(n-C_4_H_9_)_4_N][Al(OC(CF_3_)_3_)_4_] by-product has been marked with an asterisk. Cu Kα radiation, 150 K. a = 6.9518(4) Å, c = 10.4183(8) Å, V = 436.036 Å^3^, wRp = 10.86%. Black line—experimentally observed data, red line—calculated data, blue line—difference between the observed and calculated data.

[(CH_3_)_4_P]BH_4_ was prepared in reaction (3) in a nearly pure form, with only barely detectable contamination with the [(*n*-C_4_H_9_)_4_N][Al(OC(CF_3_)_3_)_4_] by-product being visible on the PXD plot, Figure 2. Due to its very low solubility in dichloromethane, the product precipitated in microcrystalline form, and a few attempts of crystallization were necessary. The protic solvents or others, like liquid SO_2_ (in which a reduction to elemental sulfur occurred) appeared to be incompatible. Interestingly, the crystals grown from 1,2-dichloroethane appeared to be tetramethylphosphonium chloride, probably due to the contaminated solvent (we did not investigate this issue further). Finally, we were able to grow crystals from anhydrous acetonitrile, which appeared sufficiently stable in the reducing environment at room temperature.

### 3.2. Crystal Structures

**Figure 3:**
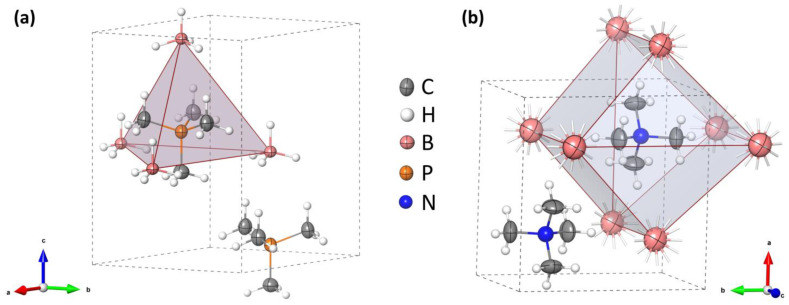
The crystal unit cell with ion coordination marked as a polyhedron for: (**a**) [(CH_3_)_4_P]BH_4_, *P*6_3_mc (this work) and (**b**) [(CH_3_)_4_N]BH_4_, *P*4/nmm (the disordered hydrogen atoms were omitted for clarity) [35,37].

Tetramethylphosphonium borohydride crystallizes in a hexagonal unit cell with Z = 2, Table 1. The structure contains both cations and anions coordinated in a slightly distorted tetrahedral geometry, with P…B = 4.1240(11)–4.143(5) Å and B…P…B = 104.39(6)–114.04(5)°, and resembles the structure of wurtzite, Figure 3a. Such a crystal structure is adopted by several salts containing [(CH_3_)_4_P]^+^, [(CH_3_)_4_As]^+^, and [(CH_3_)_4_Sb]^+^ cations and monovalent anions, like O_3_^−^, Cl^−^–I^−^, BF_4_^−^, and PF_6_^−^ [30,31,32]. On the other hand, the tetramethylammonium salts, [(CH_3_)_4_N]X, X = Cl–I, ClO_4_, BF_4_, and BH_4_, tend to crystallize in a tetragonal unit cell, in a distorted CsCl-type lattice [33,34,35], Figure 3b.

The structure of [(CH_3_)_4_P]BH_4_, despite the lack of disorder, is significantly more loosely packed than the disordered structure of [(CH_3_)_4_N]BH_4_. The molecular volume of the former is >25% larger (223.9 vs. 177.12 Å^3^ for [(CH_3_)_4_N]BH_4_ at room temperature), and its density remains ca. 6% lower despite its ca. 19% larger molecular mass. The low density of [(CH_3_)_4_P]BH_4_ is related to the voids of ca. 24 Å^3^ present in the structure (around 0 0 0.35 fractional coordinates, as listed by Olex2 [23]). Such voids might be sufficient to accommodate atoms like Ne [36]; however, no significant electron density peaks have been detected inside them.

The nearest B–H…H–C contacts of ca. 2.2 Å are shorter than the sum of the van der Waals radii of hydrogen (2.4 Å). Due to the weak polarization of the C–H^δ+^ bonds and the still substantial separation of the hydrogen atoms, these dihydrogen interactions must be of rather moderate strength. However, they are still able to fix the positions of the three hydrogen atoms of the borohydride group to remain closest to the respective hydrogen atoms of the methyl groups [38]. This is a different situation than in the case of [(CH_3_)_4_N]BH_4_, where the dihydrogen interactions with the disordered hydrogen atoms of BH_4_^−^ anions are clearly much weaker (H…H = 2.47(3) Å; a slightly shorter H…H distance of 2.40(2) Å involves the ordered H atom of the BH_4_^−^ moiety). Although the H…H contacts are shorter for [(CH_3_)_4_P]BH_4_, the distance between the adjacent heavy atoms, B…C_min_, is smaller in its ammonium analogue: 3.752(7) Å at room temperature [35], as compared to 3.833(4) Å at 100 K and 3.91(3) Å at 300 K. Obviously, the tetragonal structure of [(CH_3_)_4_N]BH_4_ facilitates better separation of B–H…H–C.

The crystal structure of [(CH_3_)_4_P]Cl shares its most important features with that of analogous borohydride, Table 1, and will not be discussed in detail. It may be noticed that the chloride expands slightly less than the borohydride when the temperature rises from 100 K to 300 K, 3.4% vs. 4.5%, respectively (a = 6.8779(2), c = 9.5941(4), V/Z = 196.53 Å^3^ for [(CH_3_)_4_P]Cl at 300 K).

### 3.3. Thermal Decomposition

**Figure 4:**
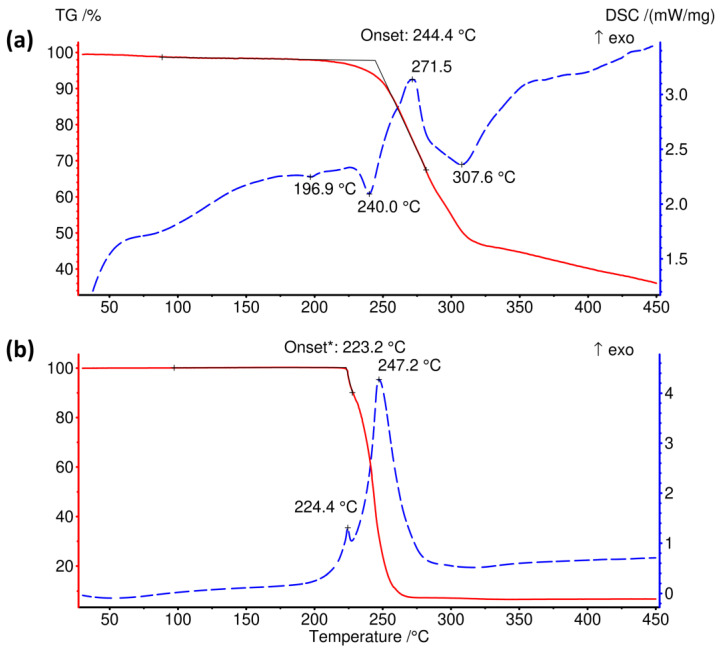
Comparison of the TGA and DSC curves of (**a**) [(CH_3_)_4_P]BH_4_ and (**b**) [(CH_3_)_4_N]BH_4_. *—onset of the first of two overlapping decomposition stages. The arrow points at the exothermic direction of DSC curves.

The thermal decomposition of [(CH_3_)_4_P]BH_4_ is preceded by an endothermic event on the DSC curve around 197 °C corresponding to 0.14 kJ mol^−1^, Figure 4. Such low heat indicates that this could be the result of a polymorphic transition or an artefact introduced by an unidentified minor contaminant.

The onset of mass loss occurs simultaneously with an endothermic DSC peak at 240 °C (1.43 kJ mol^−1^). The thermal decomposition process involves a few endo- and exothermic steps; some of them partially overlap, which is reflected by the complicated shape of the DSC curve, Figure 4a. The thermal effect of the exothermic peak at ca. 272 °C may be estimated as −8.05 kJ mol^−1^, while that of the endothermic peak at 308 °C—is 9.57 kJ mol^−1^. Although the mass loss rate significantly decreases above 320 °C, decomposition is clearly not terminated till the highest temperature of 450 °C, with the residual mass of 37.3 wt%.

The ammonium analogue of [(CH_3_)_4_P]BH_4_ decomposes in a different fashion, Figure 4b. In the case of [(CH_3_)_4_N]BH_4_, thermal decomposition occurs above ca. 223 °C in the two overlapping exclusively exothermic steps (ca. 0.7 and 53.1 kJ mol^−1^). The TGA curve flattens rather sharply above 270 °C, leaving the residual mass of only ca. 7 wt%.

### 3.4. High-Pressure Evolution of [(CH_3_)_4_P]BH_4_ as Probed by Raman Spectroscopy and DFT

**Figure 5:**
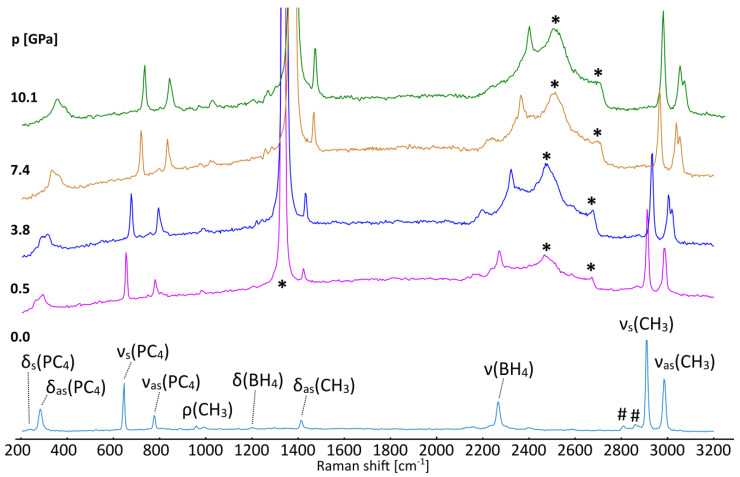
Raman spectra of [(CH_3_)_4_P]BH_4_ crystallized from acetonitrile at ambient (bottom) and elevated pressures (as listed in GPa). Identification of the most prominent vibrational modes has been shown for the ambient-pressure spectra on the basis of refs. [37,39,40]. *—the most intense signals from the diamond; #—overtones and combinatorial modes.

The Raman spectrum recorded for the recrystallized sample of [(CH_3_)_4_P]BH_4_ placed in a capillary confirms its purity, Figure 5 (bottom). No significant signals from the [(*n*-C_4_H_9_)_4_][Al(OC(CF_3_)_3_)_4_] by-product or related alkoxyaluminates were detected [18,19]. The spectrum looks as expected for the investigated compound, with it sharing the features of borohydride and tetramethylphosphonium moieties [23,37,39,40].

Under compression in a diamond anvil cell, the spectra reveal a distinct change between 0.53 and 1.86 GPa. The high-frequency ν_as_(CH_3_) mode splits, resulting in the two signals above 2990 cm^−1^, Figure 5. This change is not accompanied by any noticeable split of the other Raman peaks, which might also be biased by the limited quality of the spectra measured for the sample loaded into the DAC. A similar divergence of this mode has been reported for [(CH_3_)_4_N]BH_4_ around 5 GPa, which takes place simultaneously with a more subtle split of the δ(CH_3_) mode [37]. These changes are related to the ordering of BH_4_^−^ anions in the *P*4/nmm structure and the formation of the *P*2_1_2_1_2 phase, resembling the low-temperature behavior of [(CH_3_)_4_N]ClO_4_ [41].

The lack of significant alterations in the PC_4_ and BH_4_ bands indicates that the pressure-induced recombination of the hydridic and protic hydrogen atoms does not occur up to ca. 10 GPa. Such a process would probably require much larger pressure to achieve more pronounced compression of the B–H…H–C contacts.

**Figure 6:**
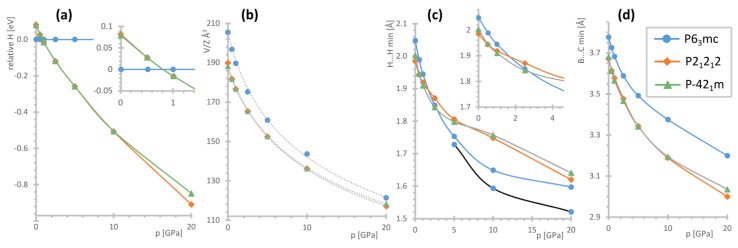
Summary of the DFT calculations for the specified structural models of [(CH_3_)_4_P]BH_4_: (**a**) relative enthalpies, (**b**) molecular volumes (points) and their fits to the Birch–Murnaghan equation (gray dotted lines), (**c**) the shortest B–H…H–C contacts; for the P6_3_mc phase, the C–H…H–C contacts are also shown using a black line; and (**d**) the shortest B…C contacts. The low-pressure regions were expanded for (**a**,**c**) for better visibility.

To gain a better insight into the possible nature of the observed phase transition, we conducted DFT calculations for several models of [(CH_3_)_4_P]BH_4_. Besides the *P*6_3_mc unit cell, the structures proposed for [(CH_3_)_4_N]BH_4_ were considered. As has been mentioned above, the ambient pressure crystal structure of the latter compound (of *P*4/nmm symmetry) reveals disorder of the BH_4_^−^ groups, cf. Figure 3, and cannot be directly optimized [35]. Therefore the two structures reported for [(CH_3_)_4_N]BH_4_ above 5 GPa (of *P*2_1_2_1_2 symmetry) and above 20 GPa (of *P*-42_1_m symmetry) were used for the calculations [37]. The selected parameters computed for the models considered here are presented in Figure 6, while the numerical data is contained in the Supplementary Materials (Table S1 and CIF files).

According to our DFT results, the *P*6_3_mc structure is the most stable at ambient pressure by ca. 0.08 eV (i.e., 7.7 kJ mol^−1^) as compared to the other models considered. The unit cell optimized with the use of dispersive corrections reveals ca. −4.1% discrepancy from the experimentally determined volume at 100 K (and ca. −8.2% from the V/Z at 300 K), which falls rather within the values expected for the accuracy of such computational methods. The optimized nearest H…H contact of 2.048 Å is 7.7% shorter than those refined to the experimental data; at the same time the computed B…C minimal distance of 3.777 Å is only ca. 1.5% shorter than the experimental one (3.833(4) Å at 100 K).

The *P*2_1_2_1_2 and *P*-42_1_m structures of considerably better packing (ca. 8% smaller molecular volume) already become energetically favored above 0.8 GPa, Figure 6a. Both these structures converge to similar geometries and are energetically indistinguishable (energy difference of around 0–2 meV) besides the highest computed pressure point, where the lower symmetry constraints of the *P*2_1_2_1_2 allow for more significant lowering of its energy. The range of pressure required for a polymorphic transition, as indicated by theoretical evidence, remains consistent with the high-pressure Raman investigation.

The theoretical high-pressure data were fitted to the third-order Birch–Murnaghan equation of state (EoS), Table 2. As could be expected, the *P*6_3_mc phase is the most compressible, although all the obtained bulk moduli remain within the typical values reported for borohydrides [36,42,43]. While the reported bulk modulus of [(CH_3_)_4_N]BH_4_ is significantly lower (5.9(6) GPa), it must be mentioned that this number was calculated for the equation optimized with a relatively high value of B_0′_ = 9.6(4). At the same time, it can be noticed that this material still shows rather similar compressibility (ca. 64% vs. 59–63% predicted for various phases of [(CH_3_)_4_P]BH_4_ within the 0–20 GPa range) [37].

Interestingly, the H…H shortest contacts and the distances between the adjacent heavy atoms, mainly B…C, evolve in a different way. The latter shows similar behavior for all the phases, with it remaining the largest for the *P*6_3_mc structure within the whole pressure range studied, Figure 6d. However, while the dihydrogen contacts for the *P*6_3_mc phase are the longest at the lowest pressure range, they shorten the most among all the models considered during compression to 5 GPa. Moreover, in this case, the C–H…H–C contacts become even shorter, Figure 6c. Consequently—it could be expected that such geometry would not be favored energetically under high pressure.

## 4. Conclusions

Tetramethylphosphonium borohydride may be successfully prepared via general ion metathesis according to Equations (2) and (3). Although the convenience of this method lies in a lack of necessity for its optimization, it requires the performance of a two-step process and the utilization of not broadly available chemicals. It appears that due to the hampered reactivity towards acetonitrile observed during the crystallization attempts, a simple, one-step reaction similar to Equation (1) might be proposed for the preparation of this compound. This remains beyond the scope of the current report, however.

[(CH_3_)_4_P]BH_4_ decomposes thermally in several endo- and exothermic steps, in strong contrast with [(CH_3_)_4_N]BH_4_, for which solely exothermic processes are observed. The onset of the mass loss of ca. 20 °C higher also indicates the slightly higher kinetic stability of the former compound.

Although [(CH_3_)_4_P]BH_4_ crystallizes in a different structure than its ammonium analogue, our experimental and theoretical results indicate that they may share the same polymorph (either the *P*2_1_2_1_2 or *P*-42_1_m structure of the related salts). One of the factors rendering the *P*6_3_mc structure relatively unstable is the proximity of the C–H…H–C contacts, which becomes unavoidable under larger pressure due to the crystallographic symmetry.

## Figures and Tables

**Table 1:** Crystallographic data for the structures reported in this work.

Compound	Tetramethylphosphonium Borohydride		Tetramethylphosphonium Chloride
Formula	[(CH_3_)_4_P]BH_4_		[(CH_3_)_4_P]Cl
T [K]	100	300	100
Space group	*P*6_3_mc	*P*6_3_mc	*P*6_3_mc
Unit cell dimensions [Å^3^]	a = 6.9190(2) c = 10.3350(3)	a = 7.0231(17) c = 10.483(3)	a = 6.8427(2) c = 9.3740(3)
c/a	1.494	1.493	1.370
V [Å^3^]	428.48(2)	447.8(2)	380.11(2)
Z	2	2	2
V/Z [Å^3^]	214.24	223.9	190.06
d [g cm^−3^]	0.821	0.786	1.106
(P–C)_min_ [Å]	1.781(3)	1.73(3)	1.777(2)
(P–C)_max_ [Å]	1.789(7)	1.761(14)	1.790(6)
(C–P–C)_min_ [°]	109.41(12)	108.1(6)	108.93(9)
(C–P–C)_max_ [°]	109.54(12)	110.8(6)	110.01(9)
(B–H)_min_ [Å]	1.186(10)	1.190(10)	-
(B–H)_max_ [Å]	1.190(10)	1.190(10)	-
(C–H)_min_ [Å]	1.079(10)	1.082(10)	1.061(7)
(C–H)_max_ [Å]	1.081(10)	1.083(10)	1.071(8)
(P…An)_min_ [Å] *	4.1240(11)	4.18(2)	3.8242(9)
(P…P)_min_ [Å]	6.53151(14)	6.6268(14)	6.12988(14)
(An…An)_min_ [Å] *	6.53151(14)	6.6268(14)	6.12988(14)
(H…H)_min_ [Å]	2.22(4)	2.16(13)	2.71(3)
Goodness of fit	1.087	1.134	1.046
R_1_ [%]	3.77	9.20	2.81
wR_2_ [%]	10.13	30.33	7.85
CSD No	2271776	2271774	2271775

* Distance involving the center of the relevant anion [An].

**Table 2:** Summary of the parameters of the Birch–Murnaghan EoS obtained for the theoretical data. The estimated standard deviations are given in parentheses. B_0′_ was refined.

Phase of [(CH_3_)_4_P]BH_4_	V_0_/Z [Å^3^]	B_0_ [GPa]	B_0′_	Max Δp [GPa]
*P*6_3_mc	205.1(22)	12.3(6)	4.3	0.55
*P*2_1_2_1_2	188.4(7)	15.0(3)	4.3	0.18
*P*-42_1_m	186.6(5)	16.2(2)	4.3	0.13

## Data Availability

The supplementary crystallographic data for this paper can be obtained free of charge from the Cambridge Crystallographic Data Centre: CCDC entries 2271774–2271777. The raw data from DFT calculations are contained in the Supplementary Materials. Other data may be provided on request by the corresponding author.

## Supplementary Materials

The following supporting information can be downloaded at: https://www.mdpi.com/article/10.3390/ma16155334/s1. Table S1: The results of the DFT calculations for [(CH_3_)_4_P]BH_4_; the optimized structures of [(CH_3_)_4_P]BH_4_ at several pressure points.
