# Supplementary material for: The Preparation, Characterization, and Pressure-Influenced Dihydrogen Interactions of Tetramethylphosphonium Borohydride
Source: Materials (Basel). 2023 Jul 29;16(15):5334. doi: 10.3390/ma16155334 (PMC10420206; doi:10.3390/ma16155334)
Supplement: Supplementary file 1 [file materials-16-05334-s001.zip › materials-2494491-supplementary.docx]

The Preparation, Characterization, and
Pressure-Influenced Dihydrogen Interactions of
Tetramethylphosphonium Borohydride

Tomasz Jaroń

Faculty of Chemistry, University of Warsaw, Pasteura 1, 02-089 Warsaw, Poland

e-mail: tjaron@uw.edu.pl

**Supplementary Information**

*Table S1. The results of DFT calculations for [(CH_3_)_4_P]BH_4_.*

| P [GPa] | a [Å] | b [Å] | c [Å] | V [Å^3^] | Z | V/Z | H | H/Z | H/Z relative |
| --- | --- | --- | --- | --- | --- | --- | --- | --- | --- |
| ***P*6_3_mc** | | | | | | | | | |
| -0.0011 | 6.843524 | – | 10.13274 | 410.9768 | 2 | 205.4884 | -2399.97379 | -1199.986895 | 0 |
| 0.509 | 6.748329 | – | 9.97976 | 393.5893 | 2 | 196.7946 | -2398.73058 | -1199.36529 | 0 |
| 0.9932 | 6.666611 | – | 9.861631 | 379.568 | 2 | 189.784 | -2397.5364 | -1198.7682 | 0 |
| 2.5003 | 6.512136 | – | 9.541795 | 350.4351 | 2 | 175.2175 | -2394.15871 | -1197.079355 | 0 |
| 4.9996 | 6.345948 | – | 9.225189 | 321.7354 | 2 | 160.8677 | -2388.96376 | -1194.48188 | 0 |
| 10.0027 | 6.139962 | – | 8.800776 | 287.3313 | 2 | 143.6656 | -2379.54821 | -1189.774105 | 0 |
| 19.9993 | 5.901873 | – | 8.045006 | 242.6815 | 2 | 121.3407 | -2363.17747 | -1181.588735 | 0 |
| ***P*6_3_mc – no dispersion correction** | | | | | | | | | |
| -0.0022 | 7.113995 | – | 10.71648 | 469.6884 | 2 | 234.8442 | -2397.94815 | -1198.974075 | – |
| ***P*2_1_2_1_2** | | | | | | | | | |
| -0.0064 | 11.56845 | 11.56256 | 5.679907 | 759.7492 | 4 | 189.9373 | -4799.6205 | -1199.905125 | 0.08177 |
| 0.5006 | 11.42814 | 11.42503 | 5.569743 | 727.2239 | 4 | 181.806 | -4797.34966 | -1199.337415 | 0.027875 |
| 1 | 11.32866 | 11.3286 | 5.50724 | 706.7874 | 4 | 176.6968 | -4795.13545 | -1198.783863 | -0.01566 |
| 2.5005 | 11.0961 | 11.08951 | 5.375659 | 661.4762 | 4 | 165.3691 | -4788.79337 | -1197.198343 | -0.11899 |
| 4.9963 | 10.80409 | 10.79681 | 5.230181 | 610.0991 | 4 | 152.5248 | -4778.95902 | -1194.739755 | -0.25788 |
| 9.9907 | 10.39599 | 10.38078 | 5.048249 | 544.7994 | 4 | 136.1998 | -4761.12058 | -1190.280145 | -0.50604 |
| 20.0089 | 9.945886 | 9.816363 | 4.795314 | 468.1781 | 4 | 117.0445 | -4729.99217 | -1182.498043 | -0.90931 |
| ***P*-42_1_m** | | | | | | | | | |
| -0.0075 | 11.54368 | – | 5.650908 | 753.0199 | 4 | 188.255 | -4799.62936 | -1199.90734 | 0.079555 |
| 0.4974 | 11.43219 | – | 5.561244 | 726.8261 | 4 | 181.7065 | -4797.34985 | -1199.337463 | 0.027828 |
| 0.9969 | 11.34106 | – | 5.496553 | 706.9644 | 4 | 176.7411 | -4795.13464 | -1198.78366 | -0.01546 |
| 2.5019 | 11.09217 | – | 5.377644 | 661.6445 | 4 | 165.4111 | -4788.791 | -1197.19775 | -0.11839 |
| 4.9998 | 10.80244 | – | 5.226946 | 609.9466 | 4 | 152.4867 | -4778.9604 | -1194.7401 | -0.25822 |
| 10.0044 | 10.38891 | – | 5.047224 | 544.7444 | 4 | 136.1861 | -4761.11774 | -1190.279435 | -0.50533 |
| 20.0017 | 9.882656 | – | 4.847602 | 473.4502 | 4 | 118.3625 | -4729.74757 | -1182.436893 | -0.84816 |

**The optimized structures of [(CH_3_)_4_P]BH_4_.**

**P = 0 GPa**

data_01_P63mc_0GPa_600

_symmetry_space_group_name_H-M 'P63MC'

_symmetry_Int_Tables_number 186

_symmetry_cell_setting hexagonal

loop_

_symmetry_equiv_pos_as_xyz

x,y,z

-y,x-y,z

-x+y,-x,z

-x,-y,z+1/2

y,-x+y,z+1/2

x-y,x,z+1/2

-y,-x,z

-x+y,y,z

x,x-y,z

y,x,z+1/2

x-y,-y,z+1/2

-x,-x+y,z+1/2

_cell_length_a 6.8435

_cell_length_b 6.8435

_cell_length_c 10.1327

_cell_angle_alpha 90.0000

_cell_angle_beta 90.0000

_cell_angle_gamma 120.0000

loop_

_atom_site_label

_atom_site_type_symbol

_atom_site_fract_x

_atom_site_fract_y

_atom_site_fract_z

_atom_site_U_iso_or_equiv

_atom_site_adp_type

_atom_site_occupancy

H2a H 0.71898 0.70203 0.58185 0 Uiso 1.00

P1 P 0.66667 0.33333 0.56113 0 Uiso 1.00

C3 C 0.66667 0.33333 0.38343 0 Uiso 1.00

B4 B 0.33333 0.66667 0.46430 0 Uiso 1.00

H4a H 0.33333 0.66667 0.58551 0 Uiso 1.00

C2 C 0.80949 0.61898 0.61955 0 Uiso 1.00

H2b H 0.80872 0.61744 0.72802 0 Uiso 1.00

H4b H 0.23548 0.47095 0.42343 0 Uiso 1.000

H3 H 0.57917 0.42083 0.34821 0 Uiso 1.00

data_01_P21212_0GPa_600eV

_symmetry_space_group_name_H-M 'P21212'

_symmetry_Int_Tables_number 18

_symmetry_cell_setting orthorhombic

loop_

_symmetry_equiv_pos_as_xyz

x,y,z

-x,-y,z

-x+1/2,y+1/2,-z

x+1/2,-y+1/2,-z

_cell_length_a 11.5685

_cell_length_b 11.5626

_cell_length_c 5.6799

_cell_angle_alpha 90.0000

_cell_angle_beta 90.0000

_cell_angle_gamma 90.0000

loop_

_atom_site_label

_atom_site_type_symbol

_atom_site_fract_x

_atom_site_fract_y

_atom_site_fract_z

_atom_site_U_iso_or_equiv

_atom_site_adp_type

_atom_site_occupancy

B1 B 0.23058 0.26292 0.16175 0 Uiso 1.00

H2 H 0.25513 0.24269 -0.04312 0 Uiso 1.00

H3 H 0.12531 0.25179 0.19039 0 Uiso 1.00

H4 H 0.28184 0.19459 0.29217 0 Uiso 1.00

H5 H 0.25901 0.36301 0.21160 0 Uiso 1.00

C6 C 0.09327 -0.08557 0.31062 0 Uiso 1.00

H7 H 0.14609 -0.02873 0.19977 0 Uiso 1.00

H8 H 0.04033 -0.14240 0.19979 0 Uiso 1.00

H9 H 0.14957 -0.13818 0.42347 0 Uiso 1.00

C10 C 0.08547 0.09317 0.67592 0 Uiso 1.00

H11 H 0.02870 0.14635 0.78612 0 Uiso 1.00

H12 H 0.13878 0.14886 0.56302 0 Uiso 1.00

H13 H 0.14154 0.04010 0.78803 0 Uiso 1.00

C14 C 0.09086 0.58695 0.29878 0 Uiso 1.00

H15 H 0.03607 0.64169 0.18738 0 Uiso 1.00

H16 H 0.14466 0.52971 0.19064 0 Uiso 1.00

H17 H 0.14637 0.64158 0.40932 0 Uiso 1.00

C18 C 0.08732 0.40919 0.66770 0 Uiso 1.00

H19 H 0.14175 0.35436 0.55556 0 Uiso 1.00

H20 H 0.03142 0.35517 0.77806 0 Uiso 1.00

H21 H 0.14190 0.46311 0.78104 0 Uiso 1.00

P22 P 0.00000 0.00000 0.49318 0 Uiso 1.00

P23 P 0.00000 0.50000 0.48323 0 Uiso 1.00

data_01_P-421m_0GPa_600eV

_audit_creation_date 2023-06-15

_symmetry_space_group_name_H-M 'P-421M'

_symmetry_Int_Tables_number 113

_symmetry_cell_setting tetragonal

loop_

_symmetry_equiv_pos_as_xyz

x,y,z

-x,-y,z

y,-x,-z

-y,x,-z

-x+1/2,y+1/2,-z

x+1/2,-y+1/2,-z

-y+1/2,-x+1/2,z

y+1/2,x+1/2,z

_cell_length_a 11.5437

_cell_length_b 11.5437

_cell_length_c 5.6509

_cell_angle_alpha 90.0000

_cell_angle_beta 90.0000

_cell_angle_gamma 90.0000

loop_

_atom_site_label

_atom_site_type_symbol

_atom_site_fract_x

_atom_site_fract_y

_atom_site_fract_z

_atom_site_U_iso_or_equiv

_atom_site_adp_type

_atom_site_occupancy

H12 H 0.13044 0.24614 0.20096 0 Uiso 1.00

C2 C 0.09355 0.91452 0.31647 0 Uiso 1.00

H1 H 0.35812 0.54047 0.20410 0 Uiso 1.00

H2 H 0.03286 0.64335 0.17980 0 Uiso 1.00

H3 H 0.36132 0.64955 0.42982 0 Uiso 1.00

H4 H 0.35659 0.96603 0.22611 0 Uiso 1.00

H7 H 0.47152 0.64673 0.20571 0 Uiso 1.00

P1 P 0.00000 0.00000 0.50000 0 Uiso 1.00

P2 P 0.50000 0.00000 0.52417 0 Uiso 1.00

H11 H 0.24436 0.74436 0.04492 0 Uiso 1.00

B B 0.26628 0.76628 0.83861 0 Uiso 1.00

H13 H 0.20582 0.70582 0.70731 0 Uiso 1.00

C1 C 0.08903 0.58903 0.29016 0 Uiso 1.00

C3 C 0.41087 0.91087 0.33844 0 Uiso 1.00

H5 H 0.14430 0.64430 0.40072 0 Uiso 1.00

H6 H 0.35610 0.85610 0.45102 0 Uiso 1.00

**P = 1 GPa**

data_P63mc_1GPa_600

_symmetry_space_group_name_H-M 'P63MC'

_symmetry_Int_Tables_number 186

_symmetry_cell_setting hexagonal

loop_

_symmetry_equiv_pos_as_xyz

x,y,z

-y,x-y,z

-x+y,-x,z

-x,-y,z+1/2

y,-x+y,z+1/2

x-y,x,z+1/2

-y,-x,z

-x+y,y,z

x,x-y,z

y,x,z+1/2

x-y,-y,z+1/2

-x,-x+y,z+1/2

_cell_length_a 6.6666

_cell_length_b 6.6666

_cell_length_c 9.8616

_cell_angle_alpha 90.0000

_cell_angle_beta 90.0000

_cell_angle_gamma 120.0000

loop_

_atom_site_label

_atom_site_type_symbol

_atom_site_fract_x

_atom_site_fract_y

_atom_site_fract_z

_atom_site_U_iso_or_equiv

_atom_site_adp_type

_atom_site_occupancy

H2a H 0.71975 0.71035 0.58217 0 Uiso 1.00

P1 P 0.66667 0.33333 0.56134 0 Uiso 1.00

C3 C 0.66667 0.33333 0.37901 0 Uiso 1.00

B4 B 0.33333 0.66667 0.46428 0 Uiso 1.00

H4a H 0.33333 0.66667 0.58843 0 Uiso 1.00

C2 C 0.81289 0.62578 0.62115 0 Uiso 1.00

H2b H 0.81232 0.62464 0.73253 0 Uiso 1.00

H4b H 0.23316 0.46633 0.42239 0 Uiso 1.00

H3 H 0.57690 0.42310 0.34295 0 Uiso 1.00

data_P21212_1GPa_600eV

_symmetry_space_group_name_H-M 'P21212'

_symmetry_Int_Tables_number 18

_symmetry_cell_setting orthorhombic

loop_

_symmetry_equiv_pos_as_xyz

x,y,z

-x,-y,z

-x+1/2,y+1/2,-z

x+1/2,-y+1/2,-z

_cell_length_a 11.3287

_cell_length_b 11.3286

_cell_length_c 5.5072

_cell_angle_alpha 90.0000

_cell_angle_beta 90.0000

_cell_angle_gamma 90.0000

loop_

_atom_site_label

_atom_site_type_symbol

_atom_site_fract_x

_atom_site_fract_y

_atom_site_fract_z

_atom_site_U_iso_or_equiv

_atom_site_adp_type

_atom_site_occupancy

B1 B 0.23354 0.26251 0.16116 0 Uiso 1.00

H2 H 0.25668 0.24294 -0.05060 0 Uiso 1.00

H3 H 0.12728 0.24665 0.19604 0 Uiso 1.00

H4 H 0.29059 0.19525 0.29198 0 Uiso 1.00

H5 H 0.25885 0.36557 0.21025 0 Uiso 1.00

C6 C 0.09606 -0.08563 0.30792 0 Uiso 1.00

H7 H 0.15041 -0.02646 0.19723 0 Uiso 1.00

H8 H 0.04283 -0.14258 0.19027 0 Uiso 1.00

H9 H 0.15281 -0.14012 0.42399 0 Uiso 1.00

C10 C 0.08557 0.09575 0.68488 0 Uiso 1.00

H11 H 0.02647 0.15011 0.79563 0 Uiso 1.00

H12 H 0.14059 0.15224 0.56930 0 Uiso 1.00

H13 H 0.14203 0.04223 0.80297 0 Uiso 1.00

C14 C 0.09175 0.58918 0.28616 0 Uiso 1.00

H15 H 0.03540 0.64472 0.17184 0 Uiso 1.00

H16 H 0.14694 0.53120 0.17452 0 Uiso 1.00

H17 H 0.14810 0.64518 0.39992 0 Uiso 1.00

C18 C 0.08896 0.40867 0.66806 0 Uiso 1.00

H19 H 0.14498 0.35246 0.55428 0 Uiso 1.00

H20 H 0.03153 0.35410 0.78199 0 Uiso 1.00

H21 H 0.14388 0.46465 0.78431 0 Uiso 1.00

P22 P 0.00000 0.00000 0.49613 0 Uiso 1.00

P23 P 0.00000 0.50000 0.47633 0 Uiso 1.00

data_P-421m_1GPa_600eV

_symmetry_space_group_name_H-M 'P-421M'

_symmetry_Int_Tables_number 113

_symmetry_cell_setting tetragonal

loop_

_symmetry_equiv_pos_as_xyz

x,y,z

-x,-y,z

y,-x,-z

-y,x,-z

-x+1/2,y+1/2,-z

x+1/2,-y+1/2,-z

-y+1/2,-x+1/2,z

y+1/2,x+1/2,z

_cell_length_a 11.3411

_cell_length_b 11.3411

_cell_length_c 5.4966

_cell_angle_alpha 90.0000

_cell_angle_beta 90.0000

_cell_angle_gamma 90.0000

loop_

_atom_site_label

_atom_site_type_symbol

_atom_site_fract_x

_atom_site_fract_y

_atom_site_fract_z

_atom_site_U_iso_or_equiv

_atom_site_adp_type

_atom_site_occupancy

H12 H 0.12952 0.24518 0.20034 0 Uiso 1.00

C2 C 0.09506 0.91343 0.31161 0 Uiso 1.00

H1 H 0.35640 0.54115 0.19540 0 Uiso 1.00

H2 H 0.03332 0.64577 0.17234 0 Uiso 1.00

H3 H 0.35903 0.65167 0.42820 0 Uiso 1.00

H4 H 0.35493 0.96624 0.21705 0 Uiso 1.00

H7 H 0.47158 0.64937 0.19871 0 Uiso 1.00

P1 P 0.00000 0.00000 0.50000 0 Uiso 1.00

P2 P 0.50000 0.00000 0.52411 0 Uiso 1.00

H11 H 0.24263 0.74263 0.05003 0 Uiso 1.00

B B 0.26545 0.76545 0.83910 0 Uiso 1.00

H13 H 0.20434 0.70434 0.70338 0 Uiso 1.00

C1 C 0.09039 0.59039 0.28545 0 Uiso 1.00

C3 C 0.40983 0.90983 0.33241 0 Uiso 1.00

H5 H 0.14647 0.64647 0.39959 0 Uiso 1.00

H6 H 0.35391 0.85391 0.44696 0 Uiso 1.00

**P = 20 GPa**

data_P63mc_20GPa_600

_symmetry_space_group_name_H-M 'P63MC'

_symmetry_Int_Tables_number 186

_symmetry_cell_setting hexagonal

loop_

_symmetry_equiv_pos_as_xyz

x,y,z

-y,x-y,z

-x+y,-x,z

-x,-y,z+1/2

y,-x+y,z+1/2

x-y,x,z+1/2

-y,-x,z

-x+y,y,z

x,x-y,z

y,x,z+1/2

x-y,-y,z+1/2

-x,-x+y,z+1/2

_cell_length_a 5.9019

_cell_length_b 5.9019

_cell_length_c 8.0450

_cell_angle_alpha 90.0000

_cell_angle_beta 90.0000

_cell_angle_gamma 120.0000

loop_

_atom_site_label

_atom_site_type_symbol

_atom_site_fract_x

_atom_site_fract_y

_atom_site_fract_z

_atom_site_U_iso_or_equiv

_atom_site_adp_type

_atom_site_occupancy

H2a H 0.71492 0.72863 0.56339 0 Uiso 1.00

P1 P 0.66667 0.33333 0.59531 0 Uiso 1.00

C3 C 0.66667 0.33333 0.37410 0 Uiso 1.00

B4 B 0.33333 0.66667 0.46765 0 Uiso 1.00

H4a H 0.33333 0.66667 0.61430 0 Uiso 1.00

C2 C 0.83446 0.66892 0.63201 0 Uiso 1.00

H2b H 0.86991 0.73981 0.75823 0 Uiso 1.00

H4b H 0.22473 0.44945 0.41611 0 Uiso 1.00

H3 H 0.56584 0.43416 0.33080 0 Uiso 1.00

data_07_P21212_20GPa_600eV

_symmetry_space_group_name_H-M 'P21212'

_symmetry_Int_Tables_number 18

_symmetry_cell_setting orthorhombic

loop_

_symmetry_equiv_pos_as_xyz

x,y,z

-x,-y,z

-x+1/2,y+1/2,-z

x+1/2,-y+1/2,-z

_cell_length_a 9.9459

_cell_length_b 9.8164

_cell_length_c 4.7953

_cell_angle_alpha 90.0000

_cell_angle_beta 90.0000

_cell_angle_gamma 90.0000

loop_

_atom_site_label

_atom_site_type_symbol

_atom_site_fract_x

_atom_site_fract_y

_atom_site_fract_z

_atom_site_U_iso_or_equiv

_atom_site_adp_type

_atom_site_occupancy

B1 B 0.23705 0.27105 0.16958 0 Uiso 1.00

H2 H 0.25097 0.25276 -0.06973 0 Uiso 1.00

H3 H 0.11880 0.27457 0.22180 0 Uiso 1.00

H4 H 0.28992 0.17874 0.29262 0 Uiso 1.00

H5 H 0.28980 0.37719 0.23195 0 Uiso 1.00

C6 C 0.09443 -0.09948 0.28013 0 Uiso 1.00

H7 H 0.15603 -0.04126 0.13865 0 Uiso 1.00

H8 H 0.01876 -0.15651 0.16505 0 Uiso 1.00

H9 H 0.15428 -0.16899 0.40144 0 Uiso 1.00

C10 C 0.08158 0.12081 0.71149 0 Uiso 1.00

H11 H 0.00065 0.18725 0.78269 0 Uiso 1.00

H12 H 0.14678 0.17675 0.57069 0 Uiso 1.00

H13 H 0.13736 0.08065 0.88566 0 Uiso 1.00

C14 C 0.10884 0.59240 0.24960 0 Uiso 1.00

H15 H 0.06231 0.65070 0.08581 0 Uiso 1.00

H16 H 0.17493 0.51365 0.16590 0 Uiso 1.00

H17 H 0.16785 0.65737 0.38412 0 Uiso 1.00

C18 C 0.11148 0.42032 0.68998 0 Uiso 1.00

H19 H 0.17804 0.35636 0.57144 0 Uiso 1.00

H20 H 0.06549 0.36539 0.85957 0 Uiso 1.00

H21 H 0.16729 0.50717 0.77256 0 Uiso 1.00

P22 P 0.00000 0.00000 0.50696 0 Uiso 1.00

P23 P 0.00000 0.50000 0.46264 0 Uiso 1.00

data_01_P-421m_20GPa_600eV

_symmetry_space_group_name_H-M 'P-421M'

_symmetry_Int_Tables_number 113

_symmetry_cell_setting tetragonal

loop_

_symmetry_equiv_pos_as_xyz

x,y,z

-x,-y,z

y,-x,-z

-y,x,-z

-x+1/2,y+1/2,-z

x+1/2,-y+1/2,-z

-y+1/2,-x+1/2,z

y+1/2,x+1/2,z

_cell_length_a 9.8827

_cell_length_b 9.8827

_cell_length_c 4.8476

_cell_angle_alpha 90.0000

_cell_angle_beta 90.0000

_cell_angle_gamma 90.0000

loop_

_atom_site_label

_atom_site_type_symbol

_atom_site_fract_x

_atom_site_fract_y

_atom_site_fract_z

_atom_site_U_iso_or_equiv

_atom_site_adp_type

_atom_site_occupancy

H12 H 0.12153 0.23628 0.23461 0 Uiso 1.00

C2 C 0.11109 0.91518 0.28351 0 Uiso 1.00

H1 H 0.36565 0.55559 0.12040 0 Uiso 1.00

H2 H 0.04049 0.66970 0.13478 0 Uiso 1.00

H3 H 0.34287 0.66638 0.40620 0 Uiso 1.00

H4 H 0.35162 0.97752 0.18147 0 Uiso 1.00

H7 H 0.49113 0.67912 0.20040 0 Uiso 1.00

P1 P 0.00000 0.00000 0.50000 0 Uiso 1.00

P2 P 0.50000 0.00000 0.54114 0 Uiso 1.00

H11 H 0.25582 0.75582 0.07333 0 Uiso 1.00

B B 0.26458 0.76458 0.83257 0 Uiso 1.00

H13 H 0.18529 0.68529 0.73363 0 Uiso 1.00

C1 C 0.10292 0.60292 0.25717 0 Uiso 1.00

C3 C 0.40574 0.90574 0.30834 0 Uiso 1.00

H5 H 0.16204 0.66204 0.40192 0 Uiso 1.00

H6 H 0.33846 0.83846 0.41424 0 Uiso 1.00
